# Granisetron as a Novel Treatment for Acute Food Protein-induced Enterocolitis Syndrome

**DOI:** 10.31662/jmaj.2025-0166

**Published:** 2025-08-22

**Authors:** Kouhei Hagino, Marei Omori, Daisuke Harama, Kotaro Umezawa, Daichi Suzuki, Chisato Jimbo, Tomoki Yaguchi, Fumi Ishikawa, Seiko Hirai, Kenji Toyokuni, Tatsuki Fukuie, Yukihiro Ohya, Kiwako Yamamoto-Hanada

**Affiliations:** 1 Allergy Center, National Center for Child Health and Development, Tokyo, Japan; 2 Department of Pediatrics, Kochi Medical School, Kochi University, Kochi, Japan; 3 Department of Occupational and Environmental Health, Graduate School of Medical Sciences, Nagoya City University, Aichi, Japan; 4 Division of General Allergy, Bantane Hospital, Fujita Health University, Aichi, Japan

**Keywords:** food protein-induced enterocolitis syndrome, granisetron, oral food challenge test

## Abstract

Acute food protein-induced enterocolitis syndrome (FPIES) is a non-immunoglobulin E-mediated food allergy characterized by delayed-onset vomiting, which can lead to severe dehydration and shock. Ondansetron, a 5-hydroxytryptamine 3 receptor antagonist, is recommended for managing acute episodes, but granisetron, a similar antiemetic, lacks global approval for FPIES. Our case series aimed to evaluate the effectiveness and safety of intravenous granisetron in acute FPIES episodes. We report three cases of infants with acute FPIES triggered by egg yolk during oral food challenges. Each patient received intravenous granisetron after multiple vomiting episodes. Clinical outcomes, including cessation of vomiting and resumption of oral intake, were assessed. Vomiting ceased within an hour after granisetron administration in all cases, allowing successful oral rehydration. No adverse effects or recurrence of symptoms were observed, and all patients were discharged the following day. The clinical outcomes were comparable to those reported for ondansetron. Our findings suggest that intravenous granisetron is a safe and effective secondary therapy for acute FPIES reactions. Given the limited evidence on granisetron for FPIES, accumulating case reports is essential for guiding clinical trials and regulatory approval. Further comparative studies of granisetron and ondansetron are warranted.

## Introduction

Acute food protein-induced enterocolitis syndrome (FPIES) is a non-immunoglobulin E-mediated food allergy with delayed vomiting 1 to 4 hours post-ingestion, without skin or respiratory symptoms ^(1), (2), (3)^. Severe cases cause hypotension, shock, or acidosis ^(4)^. The FPIES International Consensus Guidelines ^(5)^ and recent update algorithm ^(6)^ recommend intravenous fluids (normal saline 20 mL/kg) and systemic steroids to address dehydration and inflammation. However, systemic steroids are not the primary treatment owing to their delayed effect, and evidence is limited ^(7)^.

Antiemetics, specifically 5-hydroxytryptamine 3 (5-HT3) receptor antagonists, are commonly used for rapid symptom control ^(8)^. The guidelines suggest ondansetron for mild cases in children older than six months and as supportive therapy for moderate-to-severe cases. However, granisetron, another 5-HT3 antagonist, is not globally approved for FPIES. In Japan, neither is approved for FPIES, and Japanese guidelines do not include them as standard treatment. Granisetron is expected to gain approval, including in international guidelines.

Both therapies are used for vomiting from chemotherapy, radiation, and surgery. Granisetron, introduced later, has a longer half-life and has indicated superior efficacy in preventing chemotherapy-induced and postoperative nausea and vomiting (Table 1) ^(9), (10)^. However, there is a lack of evidence regarding its effectiveness for acute FPIES episodes. To address this, we administered granisetron under approval by our institution’s unauthorized-use review committee (number 2024-06), with written informed consent from caregivers. In addition, according to our institutional policy, for case reports involving three or fewer patients, a waiver of ethics committee approval is granted if consent for publication is obtained from the patients and/or their families. Because this case report involves three cases, we obtained a waiver of ethics committee approval. We report three cases of granisetron use during oral food challenges.

**Table 1.** Similarities of and Differences between Ondansetron and Granisetron.

Medications	Ondansetron	Granisetron
International FPIES guidelines	Recommended	None
Japanese FPIES guidelines	None	None
Year available	1990	1991
Mechanism of action	Blocking 5-HT3 receptors suppresses the action of serotonin, which is involved in the vomiting reflex.	
Indications	Prevention and treatment of chemotherapy-induced nausea and vomiting, vomiting after radiation therapy, and postoperative nausea and vomiting	
Side effects	The main side effects are constipation, headache, dizziness, etc. Caution is required given QT prolongation and cardiac arrhythmia may occur in rare cases.	
Half-life	Approximately 4 hours in adult patients with cancer (as per FDA data)	Approximately 9 hours after a single intravenous dose (as per FDA data)
Effect duration	It has a short half-life and may need to be given multiple times a day. It is best used to treat acute vomiting and for short-term symptom management.	There are reports of a long half-life in children. In many cases, once a day administration is sufficient. It is suitable for cases when continuous prevention of nausea and vomiting is required.
Different dosage forms	Oral tablets, dissolving tablets, intravenous injections, infusions	Oral tablets, intravenous injections, transdermal patches (long-acting)
	It is available as an oral or dissolving tablet, making it suitable for use when short-term care is required.	Depending on the patient’s condition, there are many different dosage forms: Transdermal patches are suitable when chemotherapy lasts for several days.
Metabolic pathways	Metabolized by hepatic CYP450 enzymes (mainly CYP3A4, CYP2D6).	It is metabolized primarily in the liver (CYP3A4), with little effect from CYP2D6.

5HT3: 5-hydroxytryptamine 3; FDA: Food and Drug Administration; FPIES: food protein-induced enterocolitis syndrome.

## Case Report

### Case 1: 1-year-old boy (Figure 1)

*Chief Report:* Vomiting after egg yolk ingestion

*Medical History:* Hydronephrosis

At 8 and 9 months of age, the patient experienced multiple episodes of vomiting approximately 2 hours after consuming half an egg yolk, with diarrhea. Acute FPIES was suspected, and he was referred to our hospital. At 13 months, the patient underwent an egg yolk challenge to confirm remission. Vomiting occurred 130 minutes post-ingestion, followed by frequent episodes and facial pallor. Intravenous access was secured, and granisetron was administered 240 minutes post-ingestion. Vomiting ceased, and oral fluids were resumed 330 minutes post-ingestion without further episodes. The patient tolerated subsequent meals and was discharged the next day.

**Figure 1.**
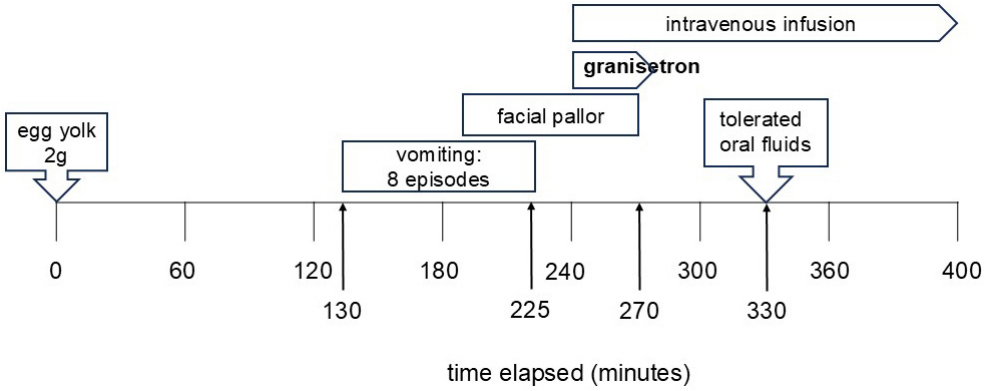
The clinical course observed during the oral challenge test in case 1, showing the steps and results of the test.

### Case 2: 1-year-old boy (Figure 2)

*Chief Report*: Vomiting after egg yolk ingestion

*Medical History*: Biliary atresia, post-liver transplantation

At 10 months, the patient experienced three episodes of vomiting 4 hours after consuming one egg yolk, followed by diarrhea. His family eliminated eggs from his diet. At 13 months, he was examined in our department, and FPIES was suspected. At 17 months, he underwent an egg yolk challenge. Vomiting occurred 190 minutes post-ingestion, with mild pallor. Intravenous fluids were initiated, and granisetron was administered 300 minutes post-ingestion. Vomiting ceased, and the patient tolerated subsequent meals without recurrence. He was discharged the next day.

**Figure 2.**
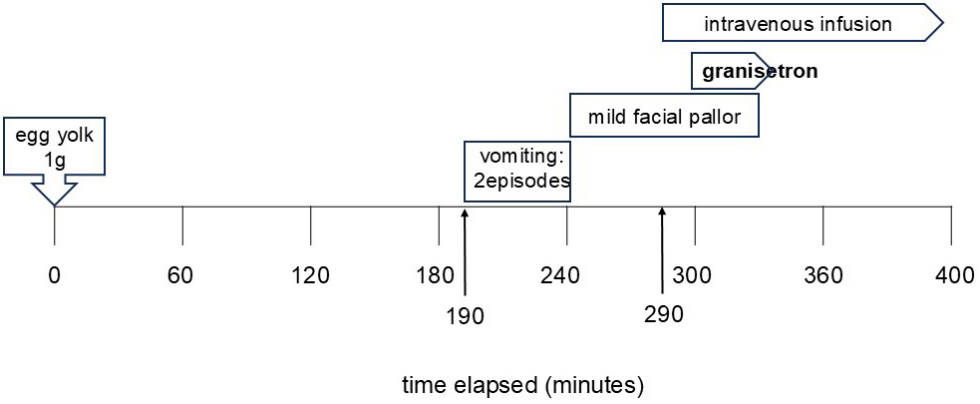
The clinical course observed during the oral challenge test in case 2, showing the steps and results of the test.

### Case 3: 1-year-old girl (Figure 3)

*Chief Report*: Vomiting after egg yolk ingestion

*Medical History*: FPIES due to soy, atopic dermatitis, developmental delay

At 7 months, the patient experienced vomiting 15 minutes to 1.5 hours after consuming half an egg yolk. At 9 months, FPIES was suspected, and she was referred to our hospital. At 13 months, an egg yolk challenge confirmed the diagnosis. At 38 months, the patient underwent another challenge. Vomiting occurred 120 minutes post-ingestion, followed by multiple episodes and facial pallor. Intravenous access was secured at 180 minutes, and granisetron was administered at 240 minutes. Vomiting ceased, and oral fluid intake resumed at 310 minutes. The patient tolerated subsequent meals and was discharged the next day.

**Figure 3.**
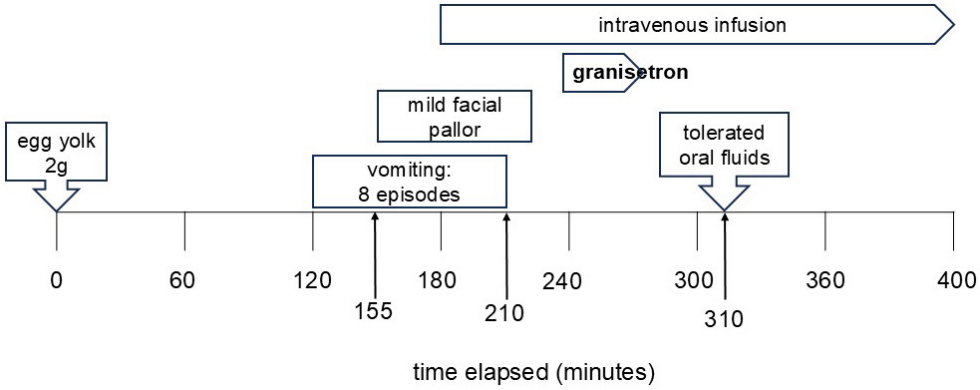
The clinical course observed during the oral challenge test in case 3, showing the steps and results of the test.

## Discussion

Intravenous granisetron was safe and effective, with vomiting cessation and oral intake resumption within an hour, similar to ondansetron outcomes. Case reports provide initial evidence of a new treatment’s efficacy and safety, particularly in off-label drug use. However, we have a few limitations to consider. First, because intravenous fluids were administered concurrently, it is difficult to attribute the observed clinical improvement solely to granisetron. On the basis of our experience, we have clearly observed that vomiting resolved much more rapidly than in previous cases. Second, the time required for drug administration was longer than usual because this was the first use of granisetron at our center, requiring additional time for preparation and coordination among the pharmacy, nursing staff, and other personnel. With increasing experience, the administration process has since become more streamlined.

Granisetron, a 5-HT3 receptor antagonist, inhibits serotonin-mediated activation of vagal afferent pathways in the gastrointestinal tract, thereby suppressing vomiting. Although the pathophysiology of FPIES remains incompletely understood, it is hypothesized that intestinal inflammation and cytokine release after antigen exposure may activate the enteric nervous system. Serotonin may contribute to vomiting in FPIES through this mechanism, similarly to its established role in chemotherapy-induced emesis ^(11)^.

Granisetron is generally well tolerated; however, several potential adverse effects have been reported. The most common side effects include headache, constipation, dizziness, and fatigue. Allergic reactions such as rash, pruritus, or, rarely, anaphylaxis may also occur. In rare cases, cardiovascular effects, including QT interval prolongation, and liver function abnormalities have been observed. Although serious adverse events are not common, careful monitoring is recommended. In the present cases, however, we did not observe any adverse events. Accumulating case reports supports clinical trial design, informing dosage, treatment efficacy measures, and potential adverse effects. Regulatory authorities, such as the Pharmaceuticals and Medical Devices Agency (PMDA) and Food and Drug Administration (FDA), may consider formal clinical trials based on case report accumulation. Our case series provides a rationale for further studies comparing granisetron and ondansetron as secondary therapies for treatment of acute FPIES.

## Article Information

### Conflicts of Interest

None

### Acknowledgement

We have obtained consent from the patients and their guardians for the presentation of these cases. This study was partially supported by the Research Fund for Development of Pediatric Medicine and the Nippon Ham Foundation.

### Author Contributions

Kouhei Hagino and Kiwako Yamamoto-Hanada established the concept of this case study. All authors followed the case in the hospital. Yukihiro Ohya, and Kiwako Yamamoto-Hanada supervised this case. Kouhei Hagino wrote the draft of the manuscript. All authors critically reviewed the manuscript and approved the final version.

### Approval by Institutional Review Board (IRB)

Not applicable. Regarding the administration of granisetron, we obtained informed consent from the patients and their families using a written informed consent document that had been approved by our institution’s unauthorized-use review committee.

In addition, according to our institutional policy, for case reports involving three or fewer patients, a waiver of ethics committee approval is granted, provided that consent for publication is obtained from the patients and/or their families. Written informed consent was obtained from the patient’s parents to use and publish clinical data.
